# Investigating the In Vitro Immunomodulatory Potential of Microparticulate β-L-Adenosine in Particulate Vaccine Candidates

**DOI:** 10.3390/vaccines14030215

**Published:** 2026-02-27

**Authors:** Snehitha Akkineni, Dedeepya Pasupuleti, Mahek Anil Gulani, Yash Harsoda, Martin J. D’Souza, Christiane Chbib, Mohammad N. Uddin

**Affiliations:** 1 College of Pharmacy, Mercer University, Atlanta, GA 30341, USA; snehitha.akkineni@live.mercer.edu (S.A.); mahekanil.gulani@live.mercer.edu (M.A.G.); yashkumar.pankajbhai.harsoda@live.mercer.edu (Y.H.); dsouza_mj@mercer.edu (M.J.D.); 2 College of Pharmacy, Larkin University, Miami, FL 33169, USA; dpasupuleti@larkin.edu; 3 Nova Southeastern University Dr Kiran C. Parel College of Allopathic Medicine (NSU-MD), Fort Lauderdale, FL 33328, USA

**Keywords:** immunomodulator, adenosine, macroparticles, antigen-presenting molecules, co-stimulatory

## Abstract

Background: Immunomodulatory compounds can modify or regulate the immune responses. Given that vaccine-induced immune responses can vary in magnitude and durability depending on antigen properties and adjuvant selection. Immunomodulators that enhance antigen-specific immune responses with low toxicity may complement existing adjuvant systems. Recent studies indicate that adenosine receptor–mediated signaling can modulate dendritic cell (DC) function through mechanisms distinct from classical pathogen-associated molecular pattern (PAMP)-driven Toll-like receptor pathways. Methods: In this context, the present study comparatively evaluates poly-(lactic-co-glycolic acid) (PLGA) microparticle–encapsulated β-L-adenosine (BLA MPs) alongside established FDA-approved adjuvants to assess their immunomodulatory potential under limited-antigen conditions. FDA-approved PLGA was used to encapsulate BLA in combination with multiple viral antigens, including H1N1 influenza, Zika virus, and canine coronavirus, to enable sustained delivery, antigen protection, and efficient uptake by antigen-presenting cells. Results: Physicochemical characterization demonstrated uniform particle size distribution, a low polydispersity index, and a stable negative surface charge. Release studies showed more than 50% payload release within 12 h, with release kinetics best described by the Korsmeyer–Peppas model. Cytotoxicity evaluation using DC2.4 cells confirmed that BLA MPs were non-cytotoxic at concentrations up to 250 μg/mL. Comparative in vitro immunological assessments revealed that BLA MPs induced dendritic cell activation, including upregulation of antigen-presenting and co-stimulatory molecules, at levels largely comparable to those observed with Alum- and MF59-based formulations across multiple antigen groups. Nitric oxide production remained within comparable ranges, indicating balanced immunostimulatory activity without excessive inflammatory signaling. In select conditions, co-formulation of BLA MPs with MF59 further enhanced DC activation, supporting its role as a complementary immunomodulatory component. Conclusion: These findings align with previously reported adenosine-dependent pathways involved in DC maturation and antigen presentation. Overall, this comparative study demonstrates that PLGA-encapsulated β-L-adenosine functions as an effective immunomodulatory agent, with performance comparable to that of established FDA-approved adjuvants across diverse vaccine antigens. Further in vivo studies are warranted to evaluate dose dependency, cytokine profiles, and antibody responses to define its role within combinatorial vaccine adjuvant strategies.

## 1. Introduction

Immunomodulation is an intervention targeting the immune system and is defined as the process by which the immune responses are modified or regulated to protect cells from various conditions. Compounds used for this purpose can be broadly classified as specific or non-specific immunomodulators. Specific immunomodulators elicit a targeted immune response, whereas the non-specific immunomodulators induce an immune response that is not directed towards a particular target [1]. Vaccines can be classified as specific immunomodulators. Vaccination results in reduced mortality, having a positive impact on public health. Although vaccines offer an excellent safety profile, they have weak antibody responses and limited Th1 responses with low immune memory, which necessitates the need for adjuvants that deliver robust and long-lasting protection. To enhance the effectiveness of vaccines and improve their immunogenicity, adjuvants were proposed [2]. During the early 1900s, vaccine adjuvants were first introduced as Alum salts, which were licensed for use in human vaccines. This was followed by the introduction of MF59, an emulsion-based adjuvant, for its registered use against the flu. The early adjuvants were mostly natural substances that progressively transitioned to synthetic compounds over time. In the 2000s, new adjuvants like AS01, AS03, and AS04 were introduced that could either be used alone or in combination [3].

Some mechanisms of action by which adjuvants enhance effectiveness include increased antigen uptake by APCs, elevated cytokine production, and the stimulation of T lymphocyte responses [4]. Adjuvants either function as immunostimulants that promote the activation of antigen-presenting cells (APCs) through specific receptor targeting or as delivery systems such as polymeric nanoparticle adjuvants, lipid-based nanoparticle adjuvants, or emulsions that aid in the antigen presentation, thereby enhancing the uptake of APCs to induce a robust immune response [5]. With adjuvants, there is a risk of increased adverse effects arising from the induction of an excessively strong immune response in addition to dose-dependent local reactions [6]. To overcome this, it was recently discovered that incorporating immunomodulators into vaccine adjuvants can better regulate the body’s response. In a recent study by Kim et al., the addition of immunomodulators to the flu vaccine and typhoid vaccine resulted in increased antibody response and reduced inflammation, respectively [7]. These findings highlight that the addition of such immunomodulators enables the development of effective and safer immunotherapies.

Adenosine is a purine nucleoside, and it plays a major role in regulating tissue function through the activation of G protein-coupled receptors like A_1_, A_2A_, A_2B_, and A_3_. These receptors are predominantly expressed on the immune cell surface. Furthermore, it acts as a modulator for both immune and inflammatory responses [8]. Through A_1_ and A_3_ receptors, adenosine enhances the activation of immune surveillance at the sites of inflammation. Via A_2A_ receptors, adenosine acts at the injury sites, driving a strong T cell response towards a T_H2_ profile. According to previous studies, it was stated that adenosine has potential as an immunomodulator and not just as a simple stimulator or suppressor [8]. Moreover, the signaling of adenosine through the A_2B_ receptor plays a major role in dendritic cell (DC) differentiation, thereby generating tolerogenic, angiogenic, and proinflammatory phenotypes, highlighting the diversity of immune activation. Slight tweaking of nucleosides improves immunomodulation, not only by inducing robust germinal center responses with neutralizing antibodies but also by shifting the adaptive immunity [9,10].

**Figure 1:**
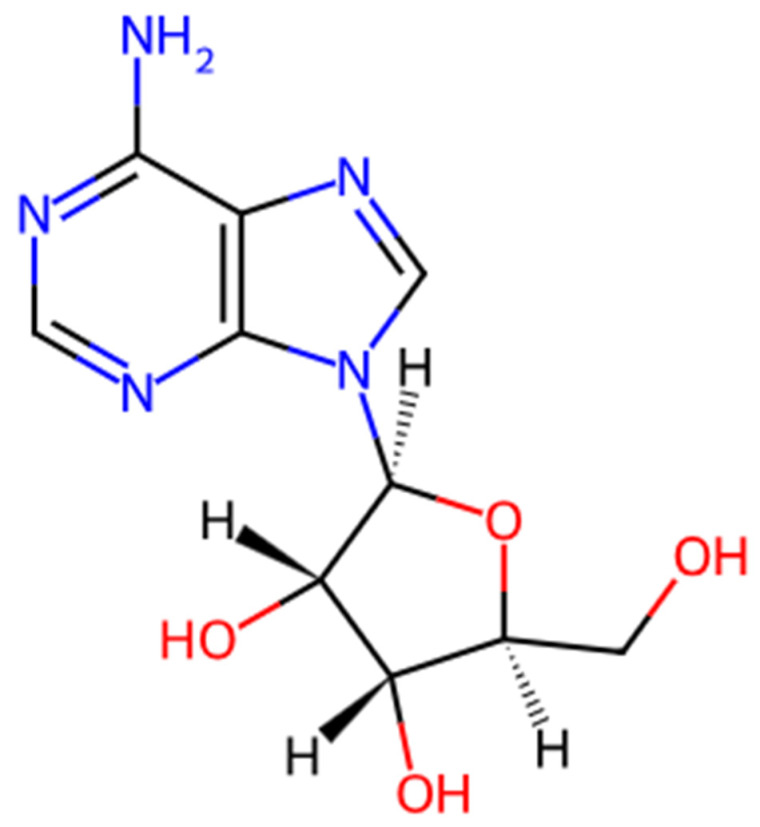
Structure of β-L-Adenosine used in this study made through ChemDraw (v25.0).

β-L-adenosine (BLA), as shown in Figure 1 (a stereoisomer of adenosine) can work as an immunomodulator by modifying innate immune signaling to improve adaptive immune responses when an antigen is co-administered. Its mechanism of action is proposed to be through purinergic (adenosine) signaling. Unlike D-adenosine, which is rapidly metabolized and predominantly immunosuppressive through A_2_ receptor signaling, β-L-adenosine exhibits enhanced metabolic stability and reduced cAMP-mediated suppression, enabling sustained APC activation and effective antigen presentation [8,11,12]. Impaired purinergic signaling often leads to neurodegenerative diseases, chronic pain, inflammatory bowel disease, and cancer. β-L-Adenosine can act on the adenosine receptors by altering the intracellular cAMP levels and thus fine-tuning the immune activation and preventing excessive inflammation [12]. They subsequently will change intracellular cAMP levels, which polishes the immune system activation instead of triggering unwarranted inflammation, and can allow antigen recognition. In turn, they enhance the DC maturation and upregulation of MHCI/II and also co-stimulatory molecules [13,14,15]. Adenosine and extracellular ATP act via P_1_ and P_2_ receptors that are expressed on most immune cells, rendering them immune response modulators [16].

In addition to the widely used nanoparticle delivery systems, polymer-based systems also have broad characteristics and applications. They exhibit high bioavailability, drug-loading, and release properties to carry vaccine antigens and immunomodulators [17,18,19]. Polymer-based systems enhance the antigen-presenting cells’ uptake through endocytosis or phagocytosis [20]. Dendritic cells play a major role in the activation of T cells, making them central to the in vitro evaluation. DC 2.4 cells can be used to compare various particle formulations and to quantify the antigen presentation in vitro [21].

Within this framework, this study aims to investigate the immunomodulatory potential of β-L-Adenosine in vitro with bacterial and viral vaccine microparticles (MPs). To achieve this, we have formulated BLA into MPs, enabling the compound to elicit a strong immune response while also providing controlled release of the antigen. The MPs were tested for their safety and immunogenicity profile. The immunomodulatory effects of BLA were evaluated with canine COVID-19, Gonorrhea, influenza, Measles, and Zika to examine how BLA will modulate the DC potential. The observed immunostimulatory responses were then compared with FDA-approved microparticulate formulations of the marketed adjuvants, like Alum and MF59, both alone and in combinations.

## 2. Materials and Methods

### 2.1. Materials

β-L-Adenosine was kindly provided by Dr. Christiane Chbib (Larkin University, Miami, FL, USA). For the double emulsion method, materials such as trehalose, polyvinyl alcohol (PVA), and dichloromethane (DCM) were obtained from Sigma-Aldrich in Burlington, VT, USA, in addition to the cell culture materials like Dulbecco’s Modified Eagle Medium (DMEM), Fetal Bovine Serum (FBS), penicillin-streptomycin, and MTT reagent-3-(4,5-dimethylthiazol-2-yl)-2,5-diphenyl tetrazolium bromide. For the in vitro study, the dendritic cell line (DC 2.4) was bought from ATCC. The polylactic-co-glycolic acid (PLGA) of 75:25 and Bovine Serum Albumin (BSA) were procured from Evonik and Fisher Scientific, respectively. Vaccine antigens like H1N1 and canine coronavirus were sourced from BEI resources. The CDC-F62 strain of Gonorrhea was a kind gift from Dr. William Shafer in GA, USA (whole cell inactivated *N. gonorrhea* vaccine antigen from the strain CDC-F62). PRVABC59, a Zika strain, was obtained from the CDC. We obtained the live attenuated Measles virus Edmonston-Zagreb strain antigen from the Serum Institute of India. Invivogen, based in San Diego, CA, USA, supplied FDA-approved adjuvants, used for this study, like MF59^®^ and Alum. Reagents used for nitrite detection through the Griess assay, like sulfanilamide, sodium nitrite, and N-1-naphthyl ethylenediamine dihydrochloride, were procured from Thermo Fisher Scientific, based in Rockford, IL, USA. All fluorescein isothiocyanate (FITC) and allophycocyanin (APC1) flow cytometry markers were purchased from eBioscience, based in San Diego, CA, USA.

### 2.2. Methods

#### 2.2.1. Formulation of Vaccine, Adjuvant, and BLA Microparticles

BLA MPs were formulated using the well-established double emulsion solvent evaporation technique [22]. Polylactic co-glycolic acid of 50 mg was dissolved in 5 mL of dichloromethane (DCM), to which 150 mL of Span 80 and 10 mg of BLA dissolved in 2 mL of PBS were also added. This solution was then subjected to homogenization using the Omni THQ probe (Kennesaw, GA, USA) at 17,000 rpm for two minutes to obtain the primary W/O emulsion. For the secondary solution, 20 mg of polyvinyl alcohol with a molecular weight of 30,000–70,000 (Sigma Aldrich, St. Louis, MO, USA) was dissolved in 20 mL of PBS. To this, the primary emulsion was slowly added. To achieve the final emulsion (W/O/W), this solution was subjected to probe homogenization at 17,000 RPM for two minutes with 30 s on and 30 s off cycles. Furthermore, DCM was evaporated with continuous stirring on a magnetic stirrer for 4 h. Subsequently, ultracentrifugation at 16,000 rpm for 20 min at 4 °C was performed to increase the microparticulate concentration. Finally, the obtained pellet was resuspended using 20 mg of trehalose in 1 mL of PBS. The emulsion containing the cryoprotectant was then lyophilized to obtain the final MPs. Additionally, adjuvants such as Alum and MF59 (FDA-approved) were also formulated using the same method.

As previously reported, inactivated H1N1 vaccine MPs were also formulated using the double-emulsion method. The heat-inactivated influenza virus strain (1%) in phosphate buffer with 7.4 pH was added to the PLGA dissolved in DCM solution at 2% *w*/*v*, followed by probe homogenization at 17,000 rpm for 2 min, forming the primary emulsion. Then, 0.1% *w*/*v* of the PVA solution in deionized water was added to the primary emulsion and again probe homogenized at the same conditions to obtain the double emulsion. DCM was evaporated from the final emulsion by placing the mixture on a plate stirrer at 500 RPM for 5 h. To obtain the concentrated microparticles, the final formulation was ultracentrifuged at 16,000 RPM for 20 min [23].

Zika MPs were also prepared using our previously published protocol. The vaccine antigen was cultured using VERO cells; they were chemically inactivated using 0.03% beta-propiolactone (BPL). The inactivated virus was purified using the centrifugal filters and used as the vaccine antigen encapsulated into the PLGA MPs using the double emulsion method as the BLA MPs [24]. Similarly, inactivated canine coronavirus MPs were formulated using the vaccine antigen in phosphate buffer with a pH of 7.4 at 1% loading. The antigen was added to PLGA in DCM 2% *w*/*v*, making it a primary emulsion through probe homogenization for 2 min at 17,000 rpm. To obtain the secondary emulsion, 0.1% PVA solution was added to the primary emulsion and was subjected to probe homogenization using the same conditions. DCM evaporation and ultracentrifugation were also performed using the same conditions as the BLA MPs for obtaining the final formulation [23].

Measles MPs were formulated using the method described in our previous publications. Spray drying technique (Buchi mini-spray dryer). Initially, 90 mg of BSA was dispersed in 5 mL of deionized water. Once the BSA was dissolved, 200 microliters of glutaraldehyde was added for every 1 g of BSA and left overnight on a magnetic stirrer at an rpm of 300, shielded from light. To this, 10 mg of sodium bisulfate was added to quench the excess glutaraldehyde added. At this stage, the overnight-prepared pre-crosslinked BSA was combined with the 5% *w*/*w* vaccine antigen. Then, 10 mL of deionized water was used to dissolve the obtained product. Spray drying was performed using a 0.5 mm nozzle at −5 °C, with an incoming temperature of 120 °C and a flow rate of 20 mL/h, with 100% aspirator operation. The formalin-inactivated whole-cell Gonorrhea vaccine antigen was used to prepare the MPs, following the same protocol described in our previous manuscripts, with a 10% loading by the spray-drying method mentioned above [25,26,27].

#### 2.2.2. Recovery Yield

All MPs obtained after lyophilization were weighed to calculate the recovery yield. Here, the weight of the microparticles is the amount of MPs collected after lyophilization. The total weight of the formulation here represents the weight before lyophilization. This was calculated using Equation (1):

Equation (1):

(1) $$Percent\;Recovery\;Yield=\frac{Weight\;of\;the\;microparticles*100}{Total\;weight\;of\;the\;formulation\;\;}\;$$

#### 2.2.3. Measurement of Particle Size, Zeta Potential, and Morphological Characterization

For the physicochemical characterization, the particle size and zeta potential of the MPs were analyzed (*n* = 4). Two milligrams of the BLA MPs were weighed and mixed with 1 mL of deionized water. Malvern Nano Zeta Sizer (Westborough, MA, USA)was used to measure the size and surface charge [28,29]. Using Scanning Electron Microscopy (SEM), the microparticles were studied for their morphology. Double-sided carbon tabs of 12 mm were attached to the stubs to which the microparticles were added and studied using the PhenomTM benchtop scanning electron microscope (Nanoscience Instruments, Phoenix, AZ, USA) [30].

#### 2.2.4. In Vitro Release Study

BLA MPs were used in an in vitro release study to determine the amount of drug released from the polymer over time. For the study, 5 mg of the MPs were dispersed in a beaker composed of 25 mL of PBS. The beaker was then transferred to a magnetic stirrer with a temperature of 37 °C and an rpm of 100. 1 mL of sample was collected from the beaker at predetermined time points, ranging from 0 to 72 h. Each time, the volume was replenished with fresh PBS post-collection. The collected samples were then subjected to centrifugation at 1500 rpm for 10 min. This was followed by the analysis of the released content at 260 nm using a UV spectrophotometer (Nanodrop 2000c—Thermo Fisher, Waltham, MA, USA).

Various kinetic models were also analyzed to study the drug release kinetics. Zero order [31] (Equation (2)) was plotted using % drug release across time, while First order [32] (Equation (3)) was plotted using % drug remaining across time. For the Higuchi model [33,34] (Equation (4)), the graph was plotted using % drug release across the square root of time, and Korsmeyer–Peppas [35,36] (Equation (5)) was plotted using the log of % drug released across the log of time.

Equation (2):

(2) $$C=k_{0}t$$

Equation (3):

(3) $$LogC_{0}-Log\;C=\;k_{1}t/2.303$$

Equation (4):

(4) $$Q=K_{H}t^{1/2}$$

Equation (5):

(5) $$M_{t}/M_{\infty }=K_{KP}t^{n}$$

All the individual variables from Equations (2)–(5) are summarized in Table 1.

#### 2.2.5. Cytotoxicity Assay

The cytotoxicity of BLA MPs was determined by the MTT (3-(4,5-dimethylthiazol-2-yl)-2,5-diphenyltetrazolium bromide) assay [37,38]. The assay was performed using murine dendritic cells (DC 2.4). DCs were plated at a seeding density of 2.8 × 10^5^ cells per well. After 24 h incubation, followed by the exposure of BLA MPs at increasing concentrations (*n* = 4), it was performed with a 100 μL volume per well. Followed by another 24 h incubation, the supernatant was removed from each well. Then, 5 mg of the MTT reagent was weighed and dissolved in 1 mL of sterile PBS to add to all wells. The plate was then protected from light and incubated for 4 h at 37 °C. After 4 h, the formazan crystals were dissolved with DMSO, and the plate was shaken on a shaker for about fifteen minutes at room temperature, shielded from light. Finally, the absorbance of the 96-well plate was measured using a plate reader (BioTek Instruments, Winooski, VT, USA) at 570 nm.

#### 2.2.6. Nitrite Quantification Through Griess Assays

The immunogenicity of the BLA MPs was assessed using the murine dendritic cells following Griess’s assay [39]. Through this assay, the nitrite released from the cells was used to study the innate immune stimulation [40]. FDA-approved adjuvant microparticulate formulations, such as Alum and MF59, along with test compounds, including BLA MPs and BLA solution, were evaluated for nitrite release. Additionally, to study the immunomodulatory effect of BLA MPs, they were combined with adjuvants, along with vaccine formulations like H1N1, Zika, Measles, Gonorrhea, and canine COVID-19. With a seeding density of 47 × 10^4^ cells per well, DCs were plated in a 48-well plate and incubated at 37 °C for 24 h. After the incubation, all experimental and control groups were incubated for 24 h. Thereafter, the supernatants from the samples were moved to another plate with 48 wells to which the Griess reagents, comprising 1% sulfanilamide in 5% phosphoric acid along with 0.1% NED (N-1-naphthylethylenediamine dihydrochloride) in deionized water, were added. Then this plate was shielded from light and incubated for 10 min, followed by the absorbance measurement at 540 nm using a plate reader (BioTek Instruments, Winooski, VT, USA). Afterwards, 100 μM of sodium nitrite was used to generate the standard curve for quantifying the nitrite concentration. Each treatment group had a different dose of MP. Every antigen, MPs, BLA MPs, and adjuvant MPs were added at 100 μg/well. Antigen + BLA/adjuvants were added at 100 μg + 50 μg/well, and lastly antigen + BLA + adjuvants were added at 100 μg + 25 μg + 25 μg/well [22,41]. The antigen-to-adjuvant ratios are mentioned in the Supplementary Table S1.

#### 2.2.7. Quantification of Antigen-Presenting Molecules Expression

DC 2.4 cells were seeded in 48-well plates and incubated for 24 h at 37 °C. On the following day, the cells were exposed to vaccine MPs, like H1N1, Zika, Measles, and COVID-19, and *N. gonorrhea* combined with BLA MPs or with FDA-approved adjuvants alone or in combinations with BLA MPs, to study the immunomodulatory effect based on the MHC I and MHC II expression, along with the control groups using the same dose as Section 2.2.6. After exposure, they were incubated for another 24 h, and then fluorescein isothiocyanate MHC I and MHC II markers (eBioscience Laboratories, San Diego, CA, USA) were used to stain the cells post-cell washing. Following this, the plate was incubated at 4 °C for 1 h, shielded from light, using a BD Accuri C6 plus flow cytometer from BD Bioscience, based in San Jose, CA, USA, and the fluorescence intensity was quantified.

#### 2.2.8. Quantification of Co-Stimulatory Molecules Expression

In a 48-well plate, DC 2.4 cells were plated and incubated for 24 h on the first day at 37 °C. On the following day, the cells were exposed to the treatment groups as mentioned in Section 2.2.7. The plate was then incubated for another 24 h. Followed by the staining with allophycocyanin (APC1) CD40 and CD80 markers (eBioscience Laboratories, San Diego, CA, USA) on day 3. Once the markers were added, the plate was shielded from light and incubated at 37 °C for 1 h. A BD Accuri C6 Plus flow cytometer from BD Biosciences, based in San Jose, CA, USA, was used to measure fluorescence intensity.

#### 2.2.9. Statistical Analysis

GraphPad Prism 10 software was used for the statistical analysis of all experiments. Mean ± Standard error of mean (SEM) was used for the illustration of the results. For the comparison of multiple experimental groups, one-way ANOVA with Tukey’s post hoc test was used. For each experiment, samples were *n* = 4. Finally, the statistical significance of the results in GraphPad was indicated as *p* > 0.05 (ns), *p* ≤ 0.05 (*), *p* ≤ 0.01 (**), *p* ≤ 0.001 (***), and *p* ≤ 0.0001 (****). Having a *p*-value less than 0.05 was treated as statistically significant, and a *p*-value greater than 0.05 was considered nonsignificant (ns).

## 3. Results

### 3.1. Formulation and Quantification of BLA Microparticles

**Figure 2:**
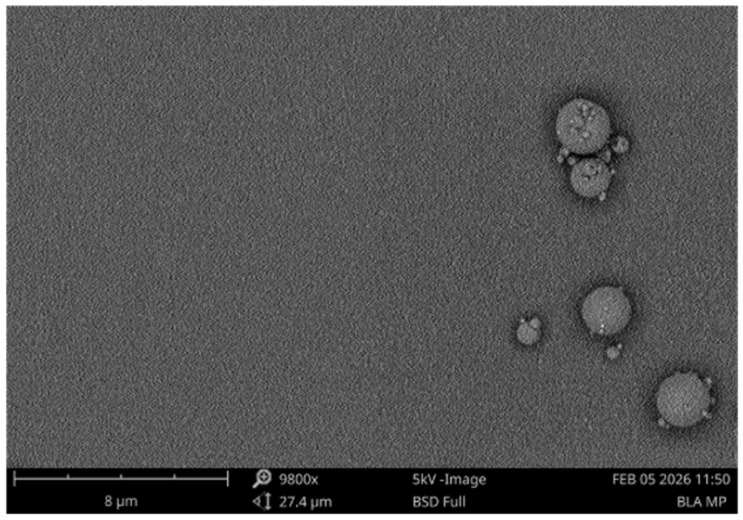
SEM image of BLA microparticles showing spherical and smooth surfaces, carried out in Phenom Benchtop SEM.

The BLA microparticles were found to have a recovery yield of 92.5%. The loss may be due to freeze-drying and high-speed centrifugation. They were analyzed in the Malvern Nano ZS, and the particle size was found to be 0.436 µm with a surface charge of −35.73 mV (Table 2). The graphical representation of the particle size is shown in Supplementary Figure S1. The morphology was observed as shown in Figure 2 using a benchtop SEM; microparticles show smooth structures with no visible deformities. We have previously demonstrated the particle sizes and encapsulation efficiencies of the vaccine MPs: canine coronavirus, with 809.2 ± 209.8 nm, with 91.7% encapsulation [23]; H1N1, with 1470 ± 108.77 nm with 92% encapsulation [42]; Zika, with 533.4 ± 9.58 nm with 55–70% encapsulation [24,43] and Gonorrhea with 3.5 µm ± 1.2 µm [27].

### 3.2. In Vitro Release Study

**Figure 3:**
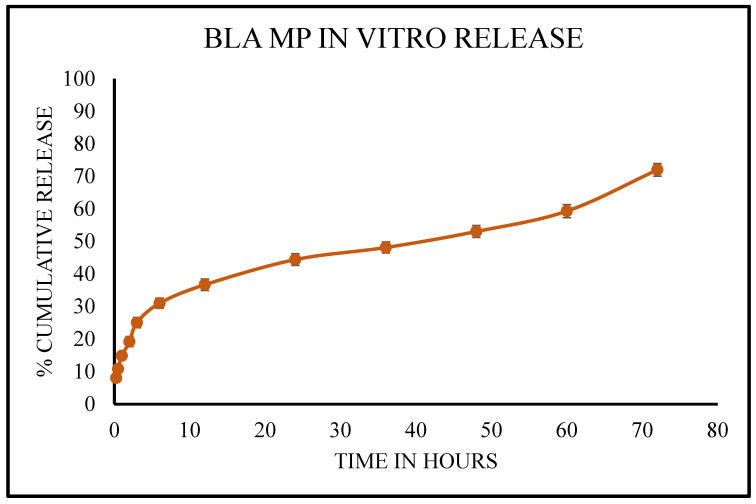
Cumulative in vitro release profile of BLA from MPs. A release study was performed in PBS at pH 7.4 at 37 °C for 72 h, indicating 50% release at approximately 8 h. Sample aliquots were taken at predetermined time points, and the volume was replaced with fresh media after sampling to maintain sink conditions. Each time point is represented as mean ± SEM, with *n* = 5.

The release study for BLA MPs was performed for 72 h. The drug release percentage from the formation was plotted across time and is shown in Figure 3. During the first 30 min, around 16% of the drug was released, and at 12 h, it increased to 59% with a burst, which was then followed by a steady release reaching 89% at the end of 72 h, showing a prolonged release of the drug from the nanoparticles, thus maintaining the effective concentration over an extended period. Phosphate-buffered saline (PBS) was chosen as the release medium based on its physiological relevance, sink condition support for drug solubility, and thus ensuring accurate cumulative release measurements through diffusion and erosion. Moreover, it is the most common release medium for analyzing the release of a drug from PLGA MPs [44].

**Figure 4:**
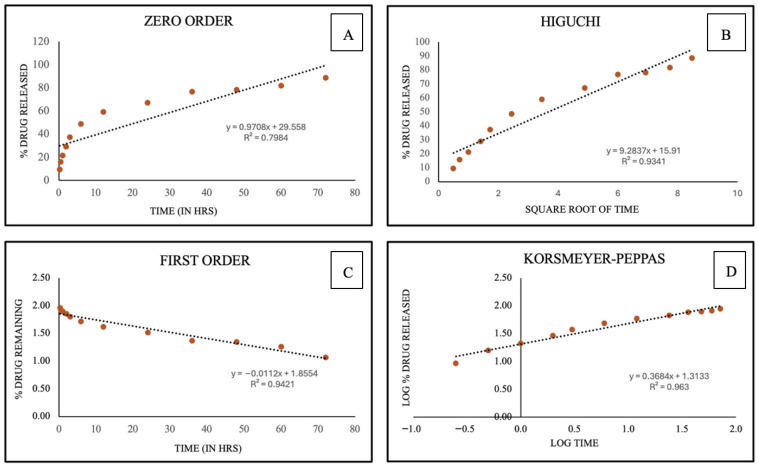
Depicts release kinetic plots: (**A**) Zero order with an R^2^ of 0.7984, (**B**) First order with an R^2^ of 0.9421, (**C**) Higuchi with an R^2^ of 0.9341, and (**D**) Korsmeyer–Peppas with an R^2^ of 0.963, *n* = 0.368.

The release study kinetics were studied and reported as shown in Figure 4, summarizing their R^2^ values. Based on the R^2^ values, the best fit was observed with the Korsmeyer–Peppas model, yielding a release exponent value (*n*) of 0.36, indicating Fickian diffusion.

### 3.3. Cytotoxicity Study

**Figure 5:**
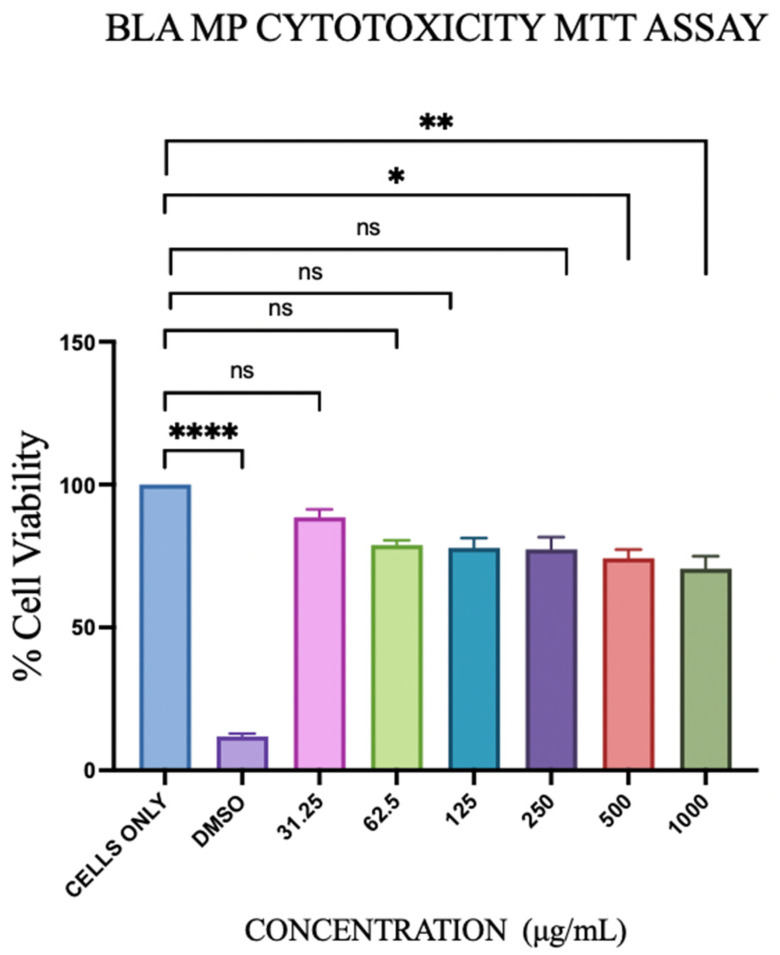
Depicts the % viability of DC 2.4 cells pulsed with BLA MPs at varying concentrations for cytotoxicity evaluation. BLA MPs did not show cytotoxicity. All groups tested are presented as mean ± SEM. Here, *p* greater than 0.05 was deemed as non-significant (ns) and *p* less than 0.05 (*), *p* ≤ 0.01 (**). The statistical significance of the results in GraphPad was indicated as *p* > 0.05 (ns), *p* ≤ 0.05 (*), *p* ≤ 0.01 (**), and *p* ≤ 0.0001 (****).

BLA MP cytotoxicity was evaluated using the DC 2.4 cells using the in vitro MTT (3-(4,5-dimethylthiazol-2-yl)-2,5-diphenyltetrazolium bromide) assay. DCs without any treatment were used as the negative control, and dimethyl sulfoxide (DMSO) was used as the positive control, as it killed the cells. BLA MPs at concentrations ranging from 31.25 μg to 1000 μg/mL were used as the treatments for cytotoxicity measurement, as shown in Figure 5. No cytotoxicity was observed from 31.25 μg to 250 μg/mL compared with cells only. Microparticles (MPs) were administered at doses of 100, 50, 25, 12.5, 6.25, and 3.12 µg per well, corresponding to BLA doses (2% loading) of 2.00, 1.00, 0.50, 0.25, 0.12, and 0.06 µg per well, respectively.

### 3.4. In Vitro Griess Assay

**Figure 6:**
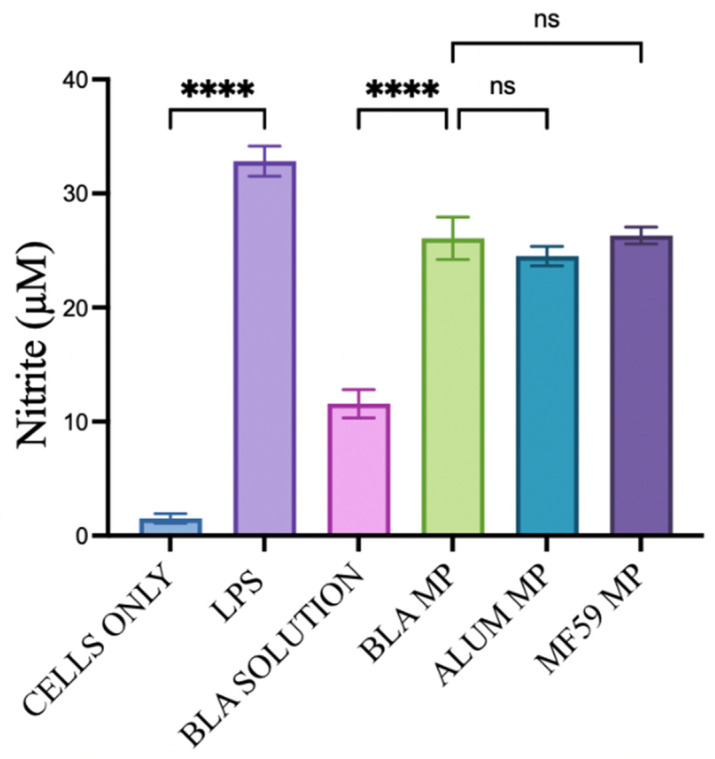
Griess assay in DC 2.4 for comparison of nitric oxide production between drug solution, drug microparticles, and marketed adjuvant microparticles (Alum and MF59). Data is presented as mean ± SEM. Here, *p* greater than 0.05 was deemed as non-significant (ns), and *p* less than 0.05 as significant. The statistical significance of the results in GraphPad was indicated as *p* > 0.05 (ns), and *p* ≤ 0.0001 (****).

When exposed to pathogens, antigen-presenting cells, including DCs, normally produce nitrate and nitrite as precursors that contribute to a robust immune response [45]. Here, it is quantified using the Griess assay with BLA MPs and FDA-approved adjuvants, combined with various bacterial and viral vaccine antigen microparticles. Figure 6 shows the nitric oxide production from the Griess assay in BLA solution, BLA MPs, Alum, and MF59. The BLA solution has a significant difference from the BLA MPs; however, there were no significant differences observed with the microparticles of the adjuvants tested.

**Figure 7:**
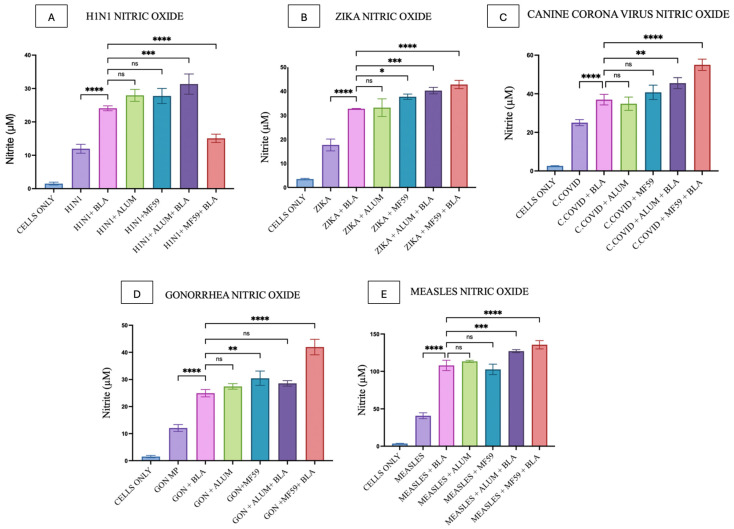
Griess assay performed in DC 2.4 cells, quantifying the nitric oxide release. (**A**) DCs treated with H1N1 MPs alone, with BLA, Alum, MF59 MPs, and in combinations. (**B**) DCs treated with Zika MPs alone, BLA, Alum, MF59 MPs, and in combinations. (**C**) DCs treated with canine COVID-19 MPs alone, BLA, Alum, MF59 MPs, and in combinations. (**D**) DCs treated with Gonorrhea MPs alone, BLA, Alum, MF59 MPs, and in combinations. (**E**) DCs treated with Measles MPs alone, BLA, Alum, MF59 MPs, and in combinations. All results are presented as mean ± SEM. The statistical significance of the results in GraphPad was indicated as *p* > 0.05 (ns), *p* ≤ 0.05 (*), *p* ≤ 0.01 (**), *p* ≤ 0.001 (***), and *p* ≤ 0.0001 (****). Having a *p*-value less than 0.05 was treated as statistically significant, and a *p*-value greater than 0.05 was considered nonsignificant (ns).

In Figure 7A, nitrite quantification from DCs pulsed with H1N1 microparticles, alone and in combination with BLA MPs, adjuvants like Alum, MF59, and combinations of these, was depicted. H1N1 MPs showed a significant difference when compared with the BLA MPs plus vaccine microparticle group, whereas there was no significant difference observed with the adjuvant MP groups. Although the H1N1 antigen, when combined with both Alum MPs and BLA MPs, elicited a higher response, the response declined when combined with MF59 MPs.

Figure 7B illustrates the notably higher response elicited when BLA MPs were combined with Zika MPs relative to Zika MPs alone. There was no significant difference between the BLA MP and Alum MP groups, whereas all other groups showed varied significant differences.

As shown in Figure 7C, the DCs were pulsed with canine COVID-19 antigen. The results indicate a significantly higher response when combined with BLA MPs, with no significant difference between the antigen adjuvant combinations and BLA MPs with the antigen. The responses were significantly lower when the antigen was combined with both BLA MP and the adjuvant MPs.

Figure 7D shows that DCs were treated with Gonorrhea MPs; a significant difference was observed when the vaccine was combined with BLA MPs. No significant difference in response was observed with the Alum adjuvant, whereas a notable difference was observed with MF59 MP. Moreover, no significant difference was observed with the Alum and BLA combination.

Finally, in Figure 7E, nitric oxide release from DCs upon treatment with Measles MPs differs significantly when the antigen was combined with BLA MPs. Similar to the previous antigen groups, BLA MPs show no significant differences with the adjuvant groups. When the antigen was treated with combinations of adjuvants and BLA MPs, responses were significantly higher than with antigen plus BLA MPs alone, indicating that the effects are better in combination.

### 3.5. Quantification of Antigen-Presenting Cells

**Figure 8:**
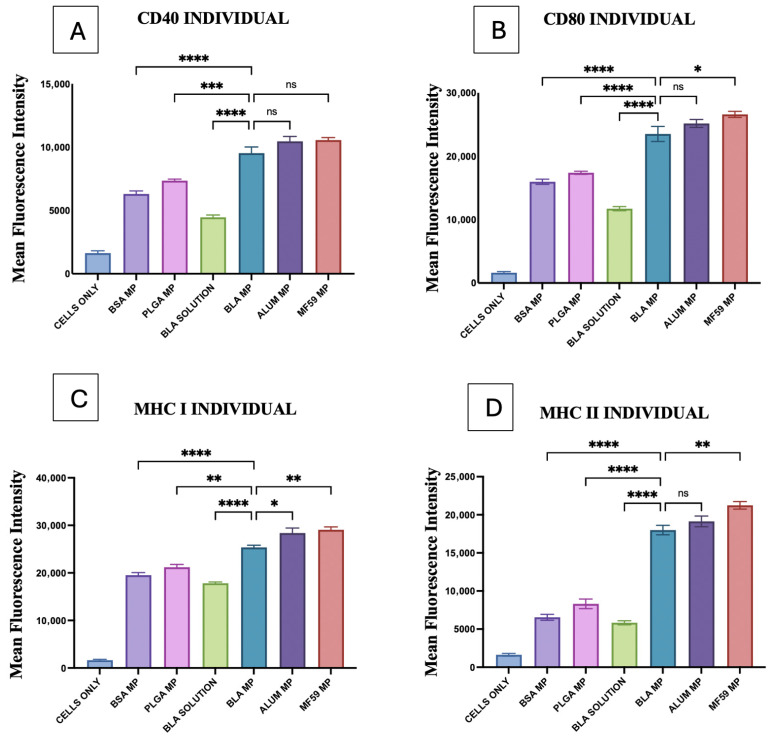
Individual comparisons of mean fluorescence intensity of all control groups: (**A**) CD40, (**B**) CD80, (**C**) MHC I, (**D**) MHC II. The results were quantified using flow cytometry after their staining with FITC markers. All the results are presented as mean ± SEM. The statistical significance of the results in GraphPad was indicated as *p* > 0.05 (ns), *p* ≤ 0.05 (*), *p* ≤ 0.01 (**), *p* ≤ 0.001 (***), and *p* ≤ 0.0001 (****). Having a *p*-value less than 0.05 was treated as statistically significant, and a *p*-value greater than 0.05 was considered nonsignificant (ns).

In Figure 8, BLA MPs were compared with blank PLGA MPs, BSA MPs, and BLA solution, along with the adjuvant MPs. Significant differences were observed between PLGA, BSA, and BLA solutions when compared with the BLA MPs. Compared with Alum MPs, a significant difference was observed only for MHC I (8C), and no significant difference was observed with MF59 in only CD40 (8A).

**Figure 9:**
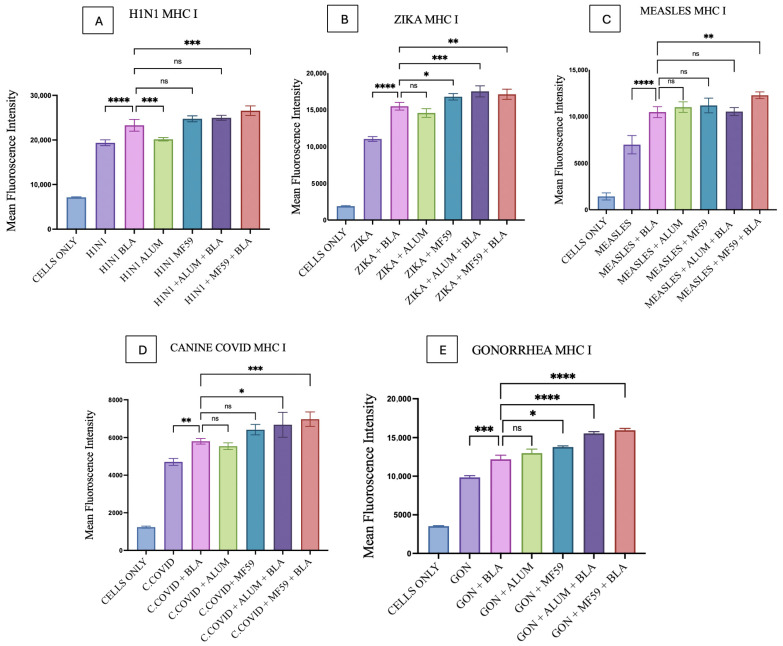
MHC I expression on DC 2.4 when pulsed with various vaccine microparticle formulations: (**A**) H1N1, (**B**) Zika, (**C**) Measles, (**D**) canine COVID-19, (**E**) Gonorrhea. The results were quantified using flow cytometry after their staining with FITC markers. All the results are presented as mean ± SEM. The statistical significance of the results in GraphPad was indicated as *p* > 0.05 (ns), *p* ≤ 0.05 (*), *p* ≤ 0.01 (**), *p* ≤ 0.001 (***), and *p* ≤ 0.0001 (****). Having a *p*-value less than 0.05 was treated as statistically significant, and a *p*-value greater than 0.05 was considered nonsignificant (ns).

**Figure 10:**
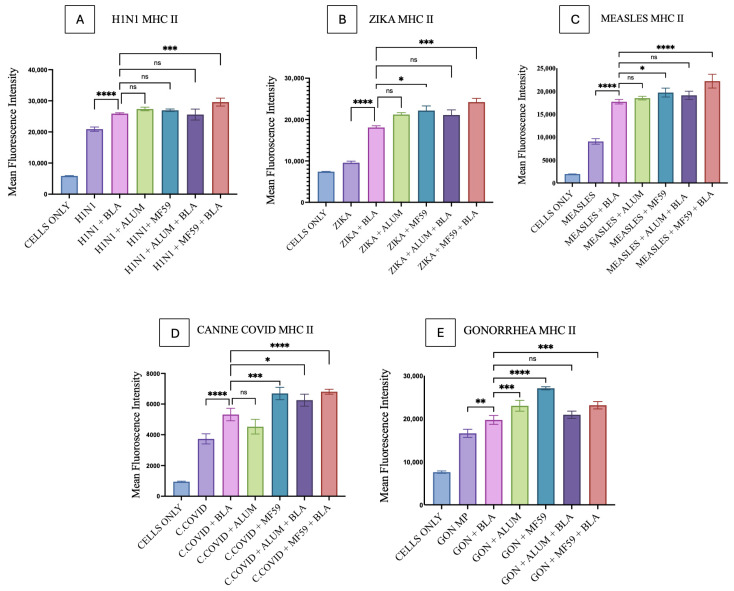
MHC II expression on DC 2.4 when pulsed with various vaccine microparticle formulations: (**A**) H1N1, (**B**) Zika, (**C**) Measles, (**D**) canine COVID-19, (**E**) Gonorrhea. The results were quantified using flow cytometry after their staining with FITC markers. All the results are presented as mean ± SEM. The statistical significance of the results in GraphPad was indicated as *p* > 0.05 (ns), *p* ≤ 0.05 (*), *p* ≤ 0.01 (**), *p* ≤ 0.001 (***), and *p* ≤ 0.0001 (****). Having a *p*-value less than 0.05 was treated as statistically significant, and a *p*-value greater than 0.05 was considered nonsignificant (ns).

MHC I and MHC II upregulation induced by BLA MPs in DC2.4 cells was analyzed using flow cytometry. All bacterial and viral vaccine antigen microparticles, such as those for Gonorrhea, H1N1, Zika, Measles, and canine COVID-19, showed a statistically significant difference in comparison with the combinations of individual antigens and BLA, as seen in Figure 9 and Figure 10. The gating strategy for all flow experiments is presented as Supplementary Figure S2.

For MHC I and MHC II expression, there was no significant difference observed when vaccine MPs combined with BLA MPs were compared with Alum MPs, except that with H1N1 vaccine microparticles, which exhibited lower levels than BLA MPs in MHC I (Figure 9A) and significantly higher levels in Gonorrhea for MHC II (Figure 10E). For MHC I, BLA MPs with vaccine MPs showed no significant difference with MF59 in H1N1 (Figure 9A), Measles (Figure 9C), and canine COVID-19 (Figure 9D) vaccine microparticles, but they did show a slight significance with Zika (Figure 9B) and Gonorrhea (Figure 9E) microparticles. Although for MHC II expression, BLA MPs with vaccine microparticles showed varied significance with all vaccine antigen MPs when compared with MF59, except with H1N1 (Figure 10A), showing no statistically significant difference.

MHC I expression of the Alum MP and BLA MP combination, when compared to the vaccine antigen with the BLA MP group, showed statistical significance in Zika, canine COVID-19, and Gonorrhea MPs, as seen in Figure 9B,D,E. Notably, the MHC II expression of the same groups with statistically significant differences was only observed in canine COVID-19 (Figure 10D) vaccine MPs. In addition, MHC I and MHC II expression on DCs was significantly higher in the MF59 MP and BLA MP combination group than in the vaccine MP and BLA MP combination groups across all vaccine MPs (Figure 9 and Figure 10). 

### 3.6. Quantification of Co-Stimulatory Molecules

**Figure 11:**
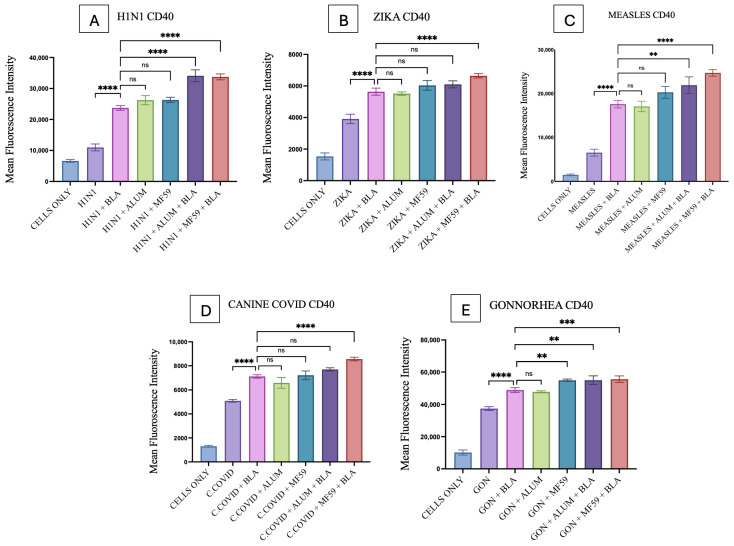
CD40 expression on DC 2.4 when pulsed with various vaccine microparticle formulations: (**A**) H1N1, (**B**) Zika, (**C**) Measles, (**D**) canine COVID-19, (**E**) Gonorrhea. The results were quantified using flow cytometry post their staining with APC markers. All the results are presented as mean ± SEM. The statistical significance of the results in GraphPad was indicated as *p* > 0.05 (ns), *p* ≤ 0.01 (**), *p* ≤ 0.001 (***), and *p* ≤ 0.0001 (****). Having a *p*-value less than 0.05 was treated as statistically significant, and a *p*-value greater than 0.05 was considered nonsignificant (ns).

**Figure 12:**
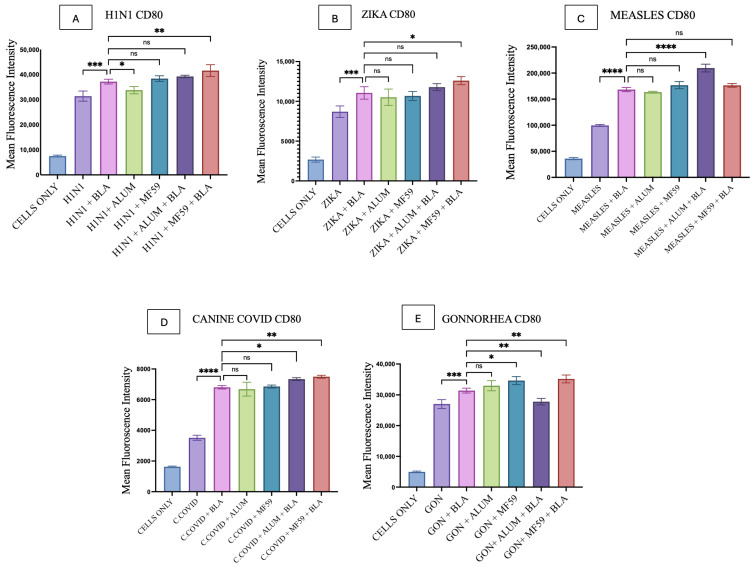
CD80 expression on DC 2.4 when pulsed with various vaccine microparticle formulations: (**A**) H1N1, (**B**) Zika, (**C**) Measles, (**D**) canine COVID-19, (**E**) Gonorrhea. The results were quantified using flow cytometry after their staining with APC markers. All the results are represented as mean ± SEM. The statistical significance of the results in GraphPad was indicated as *p* > 0.05 (ns), *p* ≤ 0.05 (*), *p* ≤ 0.01 (**), *p* ≤ 0.001 (***), and *p* ≤ 0.0001 (****). Having a *p*-value less than 0.05 was treated as statistically significant, and a *p*-value greater than 0.05 was considered nonsignificant (ns).

CD40 and CD80 upregulation induced by BLA MPs in DC2.4 cells was analyzed using flow cytometry. Combinations of individual antigens and BLA MPs, compared with vaccine microparticles alone, showed significantly higher upregulation of CD40 and CD80, as shown in Figure 11 and Figure 12. The gating strategy for all flow experiments is presented as Supplementary Figure S2.

Alum microparticles combined with all individual vaccine microparticles showed no significant differences when compared with the combinations of individual vaccine MPs with BLA MPs for both CD40 and CD80 expression, except for Alum plus H1N1 MPs, which showed a slightly lower expression in CD80 only (Figure 12A). Gonorrhea MPs combined with MF59 alone showed a significantly higher difference when compared with vaccine antigen plus BLA MPs in both CD40 and CD80 expression (Figure 11E and Figure 12E); all other vaccine MPs combined with MF59 did not show any significant difference in the same comparison (Figure 11 and Figure 12).

In addition, CD40 production in the Alum MP and BLA MP combination, compared with the BLA MP group plus vaccine MPs, was statistically significant for H1N1, Measles, and Gonorrhea MPs, as shown in Figure 11A,C,E. Similar results were observed in this comparison for CD80, but for different vaccine MPs such as Measles, canine COVID-19, and Gonorrhea (Figure 12C–E). Moreover, the CD40 and CD80 expression on DCs was significantly higher in the MF59 MP and BLA MP combination group in comparison with the vaccine MPs and BLA MPs combinations in all the vaccine MP groups (Figure 11 and Figure 12), except for Measles CD80 (Figure 12C), in the same comparison showing no significance.

## 4. Discussion

With rapidly evolving antigens, there is a need for vaccine formulations to elicit durable immune responses. To maintain this, there is a need for novel immunomodulators that elicit robust responses while also boosting antibody titers when the existing adjuvant weakens. Agents that enhance the immune responses to specific antigens with low toxicity can be classified as immunomodulators. Lately, adenosine receptor targeting has shown promising results, with immunomodulatory effects on dendritic cells, distinguishing it from the classic pathways where the DC activation is triggered by pathogen-associated molecular pathways (PAMPs) through pattern recognition receptors, specifically, like Toll-like receptors that generate responses [46,47,48]. Based on this rationale, this study aimed to test PLGA microparticle-loaded Beta-L-Adenosine for its ability to function as an immunomodulator, even at low antigen doses, using in vitro assays across a broad range of antigens. To aid in sustained delivery and cargo protection, PLGA, an FDA-approved copolymer for drug delivery, was chosen to encapsulate H1N1, Zika, and canine corona vaccine MPs and BLA MPs [49,50,51]. The use of PLGA enhances lymph node targeting, improves phagocytosis, and promotes DC presentation, resulting in sustained immunotherapeutic effects [52].

Upon characterization, the MPs revealed low PDI and a negative surface charge (Table 2). Low PDI of PLGA MPs indicates a uniform size distribution in the formulation. Additionally, because the surface charge is negative, the MPs are more stable and do not aggregate [53,54]. Moreover, the particle size is essential for the interaction with antigen-presenting cells and immune responses. DCs uptake the particles through various mechanisms; nanoparticles and microparticles ranging from 50 nm to 5 μm undergo phagocytosis or micropinocytosis. MHC I upregulation and CD86 expression on murine bone marrow dendritic cells were better presented with smaller particle sizes [29,55,56]. We acknowledge that phagocytosis is efficient with particles ranging between 1 and 3 μm, as mentioned above, but smaller particles are also efficiently internalized by the DCs and can be seen with our formulation, which produced significant immunomodulatory effects even with bacterial antigens upon co-administration [57,58].

The release study showed that more than 50% of the drug was released by 12 h (Figure 3), and based on the release kinetics studied, it can be concluded that the best fit model was observed as Korsmeyer–Peppas, a semi-empirical model with no complex interactions, and is plotted between log % cumulative released vs. log time as it has the highest R^2^ value [59].

As BLA MPs are being tested for the first time, it is necessary to evaluate their cytotoxicity. We have used DC 2.4 cells and treated them with varying concentrations of the nanoparticles and observed that they are non-cytotoxic up to 250 μg/mL and have only shown a slight difference with increasing concentrations of 500 μg/mL and 1000 μg/mL. Based on these results, we determined the dose for further in vitro studies to be lower than 250 μg. The concentrations for cytotoxicity testing were assessed based on the doses used in in vivo studies for immunomodulatory compounds [46,60,61].

Nitric oxide produced by DCs promotes DC maturation and antigen presentation. As soon as APCs recognize antigens or pathogens, immune markers are released, leading to nitric oxide production, and several immune cells are recruited to generate a strong immune response against the pathogen [62]. In this study, nitric oxide released by DC 2.4 cells was measured through the Griess assay. BLA MPs showed no significant difference in nitrite quantified when compared with both Alum and MF59 (Figure 6 and Figure 7). The quantified nitric oxide released serves as a pre-marker for determining the compound’s immunogenicity.

Through Figure 8, Figure 9, Figure 10, Figure 11 and Figure 12 (the gating strategy for all flow experiments is presented as Supplementary Figure S2), BLA MPs with vaccine MPs have shown dendritic cell activation through the expression and upregulation of antigen-presenting and costimulatory molecules. The vaccine MPs with BLA MPs’ activity were mostly comparable in both MF59 MPs and Alum MPs across all antigens, showing varied differences in only certain scenarios. However, the combination of vaccine MPs and BLA with MF59 has always shown increased expression, stating its potential to promote responses alone and in combinations as well. These findings together support that the compound demonstrates its immunostimulatory and immunomodulatory effects. Although all vaccine formulations employed BLA microparticles as a common adjuvant, distinct MHC class I and class II responses were observed across H1N1, canine coronavirus, Measles, and Gonorrhea antigens. This variability is consistent with well-established antigen-intrinsic differences in structure, stability, and innate immune signaling capacity, which collectively influence intracellular processing pathways and antigen-presenting cell (APC) subset engagement. While BLA microparticles enhance antigen uptake and APC activation, they do not override antigen-specific trafficking and processing mechanisms; thus, immune responses remain appropriately tailored to the biological characteristics of each pathogen.

Importantly, the comparisons in this study were designed to evaluate the enhancement of immunostimulatory responses conferred by BLA microparticles relative to vaccine microparticles alone, rather than to equate immune outcomes across different antigens. In our previous studies [23,43,63,64,65], particulate vaccine candidates demonstrated significantly improved immunostimulatory responses when combined with FDA-approved adjuvants in their particulate form. Based on these previous observations, the present work investigates the immunostimulatory potential of particulate BLA, both alone and in combination with the same FDA-approved adjuvants, to outline its contribution to immune activation within established particulate vaccine platforms. DCs, when differentiated, also show a distinct immunomodulatory state with higher MHC II and CD86, with lower IL-12 and higher IL-10 [66]. High concentrations of Adenosine triphosphate are said to upregulate MHC I and MHC II levels, along with the co-stimulatory molecules. This upregulation is inhibited by CD39 and CD73, as well as the adenosine P_1_ receptor antagonist, through the metabolization of ATP to adenosine. These findings suggest that ATP and adenosine are essential for the upregulation of MHC I and MHC II [67]. According to another study, adenosine deaminase, when anchored to the DC surface through the A_2B_ receptor, binds to the CD26 on T-cells, which triggers the co-stimulation. There was also an increased expression of CD80, CD83, and CD86 when compared with immature DCs [68]. Based on the study by Casanova V et al., adenosine deaminase enhanced the expression of CD80, CD83, CD86, and CD40 on immature dendritic cells from healthy and HIV-infected individuals. Here, it acts as a bridge between A_2B_ receptors on DCs and enhances the T-cell proliferation [69]. All these results are similar to those of BLA MPs, which enhance DC activation and function through adenosine receptor-dependent pathways.

The current study focuses on the DC surface marker maturation in vitro immunogenicity profile of the MPs determined through the Griess assay. One of the limitations of this study is that the cytokine profiling (i.e., IL-6, IL-12, IFN-γ) was not evaluated. Although our study observed DC upregulation with increased MHC I, MHC II, CD40, and CD80, which align with the previous reports, direct validation using the receptor antagonists and the cAMP quantification was beyond the study’s scope. Moreover, the use of the DC 2.4 cell line may not represent the entire functional spectrum of the primary dendritic cells.

Future work of this study includes several stages, such as cytokine profiling to elucidate the mechanistic pathways by which BLA acts as an immunostimulatory immunomodulator, as well as studies focusing on its anti-inflammatory and receptor-specific immunomodulatory mechanisms. Additionally, future studies will be performed with receptor antagonists to confirm the precise signaling pathways that are involved. Antigen presentations and DC upregulation will also be studied in primary dendritic cells. It is also necessary to conduct a concentration-dependent in vivo study of the BLA formulation to evaluate antibody titers and immunogenicity and provide deeper insights into safety and the immune profile. Overall, based on the results obtained, this formulation at the development stage has demonstrated potential as a pronounced immunomodulatory candidate in tested bacterial and viral vaccine antigen microparticles.

## 5. Conclusions

In the current study, BLA was formulated as microparticles with the PLGA polymer; physical characterization depicted desirable size, surface charge, PDI, and drug release. They were non-cytotoxic to dendritic cells. In vitro characterization of this compound shows potential as an immunomodulator compared with various vaccine candidates and adjuvants, and it has upregulated antigen-presenting and costimulatory molecules. Further studies are warranted to test the in vivo efficacy of this formulation.

## Figures and Tables

**Table 1:** Summary of all the variables and their definitions for the release kinetic models.

Variable	Definition
*C*	Concentration of drug at time t
*t*	Time
*K*_0_	Zero order rate constant
*C*_0_	Initial drug concentration
*k*_1_	First order rate constant
*K_H_*	Higuchi dissolution constant
*Q*	Amount of drug released
*M_t_*	Drug released at time t
*M* *_∞_*	Amount of total drug available for release
*K*	Rate constant for release
*n*	Exponent for release

**Table 2:** Physicochemical characterization of BLA MPs particle size in nanometers, polydispersity index, % recovery yield, and surface charge. (*n* = 4).

Parameter	Result
Percent yield	92.5 ± 2.5%
Particle size	0.436 ± 24.6 µm
Zeta potential	−35.73 ± 4.42 mV
Polydispersity index	0.455 ± 0.16

## Data Availability

Data available on request from the corresponding author.

## Supplementary Materials

The following supporting information can be downloaded at: https://www.mdpi.com/article/10.3390/vaccines14030215/s1, Figure S1: Graphical representation of particle size of BLA MP. Table S1: Dose in micrograms per well in each treatment groups. Figure S2: Flow cytometry gating strategy for all experiments in this study. Figure S2.A. Scatter plot showing side scattering (SSC-A) and forward scattering (FSC-A) of the cells that are selected based on their size and granularity. The selection (R1) includes 22% of events from a given sample to analyze the fluorescence intensity. Figure S2.B. shows the histogram of FITC-A fluorescence intensity of a given sample. The events are divided into left and right panel where left indicates lower FITC-A expression and right indicates higher FITC-A expression in the selected population. Figure S2.C. shows the histogram of APC-A fluorescence intensity of a given sample. The events are divided into left and right panel where left indicates lower APC-A expression and right indicates higher APC-A expression in the selected population.

## References

[1] Costa E., Machado M., Pintado M., Silva S. (2022). Biological macromolecules as immunomodulators. Biological Macromolecules.

[2] Cui Y., Ho M., Hu Y., Shi Y. (2024). Vaccine adjuvants: Current status, research and development, licensing, and future opportunities. J. Mater. Chem. B.

[3] Facciolà A., Visalli G., Laganà A., Di Pietro A. (2022). An Overview of Vaccine Adjuvants: Current Evidence and Future Perspectives. Vaccines.

[4] Xing J., Zhao X., Li X., Fang R., Sun M., Zhang Y., Song N. (2025). The recent advances in vaccine adjuvants. Front. Immunol..

[5] Lim Y.T. (2015). Vaccine adjuvant materials for cancer immunotherapy and control of infectious disease. Clin. Exp. Vaccine Res..

[6] Petrovsky N. (2015). Comparative Safety of Vaccine Adjuvants: A Summary of Current Evidence and Future Needs. Drug Saf..

[7] Kim J.Y., Rosenberger M.G., Chen S., Ip C.K., Bahmani A., Chen Q., Shen J., Tang Y., Wang A., Kenna E. (2023). Discovery of New States of Immunomodulation for Vaccine Adjuvants via High Throughput Screening: Expanding Innate Responses to PRRs. ACS Cent. Sci..

[8] Haskó G., Linden J., Cronstein B., Pacher P. (2008). Adenosine receptors: Therapeutic aspects for inflammatory and immune diseases. Nat. Rev. Drug Discov..

[9] Pardi N., Hogan M.J., Naradikian M.S., Parkhouse K., Cain D.W., Jones L., Moody M.A., Verkerke H.P., Myles A., Willis E. (2018). Nucleoside-modified mRNA vaccines induce potent T follicular helper and germinal center B cell responses. J. Exp. Med..

[10] Bernard M.-C., Bazin E., Petiot N., Lemdani K., Commandeur S., Verdelet C., Margot S., Perkov V., Ripoll M., Garinot M. (2023). The impact of nucleoside base modification in mRNA vaccine is influenced by the chemistry of its lipid nanoparticle delivery system. Mol. Ther.—Nucleic Acids.

[11] Xing J., Zhang J., Wang J. (2023). The Immune Regulatory Role of Adenosine in the Tumor Microenvironment. Int. J. Mol. Sci..

[12] Mardanyan S., Karapetyan L., Antonyan A. (2025). Adenosine and Adenosine Deaminase Contrary Manifestations in Immunity. Scand. J. Immunol..

[13] Burnstock G., Pelegrín P. (2020). Introduction to Purinergic Signaling. Purinergic Signaling.

[14] Huang Z., Xie N., Illes P., Di Virgilio F., Ulrich H., Semyanov A., Verkhratsky A., Sperlagh B., Yu S.-G., Huang C. (2021). From purines to purinergic signalling: Molecular functions and human diseases. Signal Transduct. Target. Ther..

[15] Eltzschig H.K., Sitkovsky M.V., Robson S.C. (2012). Purinergic Signaling during Inflammation. N. Engl. J. Med..

[16] Di Virgilio F., Vuerich M. (2015). Purinergic signaling in the immune system. Auton. Neurosci..

[17] Danhier F., Ansorena E., Silva J.M., Coco R., Le Breton A., Préat V. (2012). PLGA-based nanoparticles: An overview of biomedical applications. J. Control Release.

[18] Sarkar C., Kommineni N., Butreddy A., Kumar R., Bunekar N., Gugulothu K., Jana S., Jana S. (2022). PLGA Nanoparticles in Drug Delivery. Nanoengineering of Biomaterials.

[19] Omidian H., Wilson R.L., Castejon A.M. (2025). Recent Advances in Peptide-Loaded PLGA Nanocarriers for Drug Delivery and Regenerative Medicine. Pharmaceuticals.

[20] Li M., Li Y., Li S., Jia L., Wang H., Li M., Deng J., Zhu A., Ma L., Li W. (2022). The nano delivery systems and applications of mRNA. Eur. J. Med. Chem..

[21] Lu L., Kong W.Y., Zhang J., Firdaus F., Wells J.W., Stephenson R.J., Toth I., Skwarczynski M., Cruz J.L.G. (2024). Utilizing murine dendritic cell line DC2.4 to evaluate the immunogenicity of subunit vaccines in vitro. Front. Immunol..

[22] Shah S.M., Joshi D., Chbib C., Roni M.A., Uddin M.N. (2023). The Autoinducer N-Octanoyl-L-Homoserine Lactone (C8-HSL) as a Potential Adjuvant in Vaccine Formulations. Pharmaceuticals.

[23] Vijayanand S., Patil S., Joshi D., Menon I., Gomes K.B., Kale A., Bagwe P., Yacoub S., Uddin M.N., D’souza M.J. (2022). Microneedle Delivery of an Adjuvanted Microparticulate Vaccine Induces High Antibody Levels in Mice Vaccinated against Coronavirus. Vaccines.

[24] Kale A., Joshi D., Menon I., Bagwe P., Patil S., Vijayanand S., Gomes K.B., D’SOuza M. (2022). Novel microparticulate Zika vaccine induces a significant immune response in a preclinical murine model after intramuscular administration. Int. J. Pharm..

[25] Shastri P.N., Kim M., Quan F., D’Souza M.J., Kang S. (2012). Immunogenicity and protection of oral influenza vaccines formulated into microparticles. J. Pharm. Sci..

[26] Ubale R.V., D’souza M.J., Infield D.T., McCarty N.A., Zughaier S.M. (2013). Formulation of meningococcal capsular polysaccharide vaccine-loaded microparticles with robust innate immune recognition. J. Microencapsul..

[27] Gala R.P., Zaman R.U., D’Souza M.J., Zughaier S.M. (2018). Novel Whole-Cell Inactivated Neisseria Gonorrhoeae Microparticles as Vaccine Formulation in Microneedle-Based Transdermal Immunization. Vaccines.

[28] Awate S., Babiuk L.A., Mutwiri G. (2013). Mechanisms of Action of Adjuvants. Front. Immunol..

[29] Oyewumi M.O., Kumar A., Cui Z. (2010). Nano-microparticles as immune adjuvants: Correlating particle sizes and the resultant immune responses. Expert. Rev. Vaccines.

[30] Joshi D., Chbib C., Uddin M.N., D’Souza M.J. (2021). Evaluation of Microparticulate (S)-4,5-Dihydroxy-2,3-pentanedione (DPD) as a Potential Vaccine Adjuvant. AAPS J..

[31] Dash S., Murthy P.N., Nath L., Chowdhury P. (2010). Kinetic modeling on drug release from controlled drug delivery systems. Acta Pol. Pharm..

[32] Costa P., Sousa Lobo J.M. (2001). Modeling and comparison of dissolution profiles. Eur. J. Pharm. Sci..

[33] Higuchi T. (1963). Mechanism of sustained-action medication. Theoretical analysis of rate of release of solid drugs dispersed in solid matrices. J. Pharm. Sci..

[34] Medarametla R.T., Gopaiah K.V., Kumar J.N.S., Babu G.A., Shaggir M., Raghavendra G., Reddy D.N., Venkamma B. (2024). Drug Release Kinetics and Mathematical Models. Int. J. Sci. Res. Methodol..

[35] Korsmeyer R.W., Gurny R., Doelker E., Buri P., Peppas N.A. (1983). Mechanisms of solute release from porous hydrophilic polymers. Int. J. Pharm..

[36] Merchant H.A., Shoaib H.M., Tazeen J., Yousuf R.I. (2006). Once-daily tablet formulation and in vitro release evaluation of cefpodoxime using hydroxypropyl methylcellulose: A technical note. AAPS PharmSciTech.

[37] Kang K., Lim J.-S. (2012). Induction of Functional Changes of Dendritic Cells by Silica Nanoparticles. Immune Netw..

[38] Bahuguna A., Khan I., Bajpai V.K., Kang S.C. (2017). MTT assay to evaluate the cytotoxic potential of a drug. Bangladesh J. Pharmacol..

[39] Gala R.P., D’Souza M., Zughaier S.M. (2016). Evaluation of various adjuvant nanoparticulate formulations for meningococcal capsular polysaccharide-based vaccine. Vaccine.

[40] Schmölz L., Wallert M., Lorkowski S. (2017). Optimized incubation regime for nitric oxide measurements in murine macrophages using the Griess assay. J. Immunol. Methods.

[41] Gulani M., Harsoda Y., Arte T., D’souza M.J., Bagwe P., Adediran E., D’souza N., Pasupuleti D. (2025). Evaluation of Polymyxin B as a Novel Vaccine Adjuvant and Its Immunological Comparison with FDA-Approved Adjuvants. Vaccines.

[42] Singh R., Gulani M., Vijayanand S., Arte T., Adediran E., Pasupuleti D., Patel P., Ferguson A., Uddin M., Zughaier S.M. (2025). An intranasal quadruple variant vaccine approach using SARS-CoV-2 and influenza A: Delta, Omicron, H1N1and H3N2. Int. J. Pharm..

[43] Shah S., Patel P., Ferguson A., Bagwe P., Kale A., Adediran E., Singh R., Arte T., Pasupuleti D., Uddin M.N. (2024). Buccal Administration of a Zika Virus Vaccine Utilizing 3D-Printed Oral Dissolving Films in a Mouse Model. Vaccines.

[44] Shah S.R., Henslee A.M., Spicer P.P., Yokota S., Petrichenko S., Allahabadi S., Bennett G.N., Wong M.E., Kasper F.K., Mikos A.G. (2014). Effects of Antibiotic Physicochemical Properties on Their Release Kinetics from Biodegradable Polymer Microparticles. Pharm. Res..

[45] Cinelli M.A., Do H.T., Miley G.P., Silverman R.B. (2020). Inducible nitric oxide synthase: Regulation, structure, and inhibition. Med. Res. Rev..

[46] Monticone G., Huang Z., Hewins P., Cook T., Mirzalieva O., King B., Larter K., Miller-Ensminger T., Sanchez-Pino M.D., Foster T.P. (2024). Novel immunomodulatory properties of adenosine analogs promote their antiviral activity against SARS-CoV-2. EMBO Rep..

[47] Verma S.K., Mahajan P., Singh N.K., Gupta A., Aggarwal R., Rappuoli R., Johri A.K. (2023). New-age vaccine adjuvants, their development, and future perspective. Front. Immunol..

[48] Cheng H., Chen W., Lin Y., Zhang J., Song X., Zhang D. (2023). Signaling pathways involved in the biological functions of dendritic cells and their implications for disease treatment. Mol. Biomed..

[49] Silva A.L., Soema P.C., Slütter B., Ossendorp F., Jiskoot W. (2016). PLGA particulate delivery systems for subunit vaccines: Linking particle properties to immunogenicity. Hum. Vaccines Immunother..

[50] Allahyari M., Mohit E. (2016). Peptide/protein vaccine delivery system based on PLGA particles. Hum. Vaccines Immunother..

[51] Makadia H.K., Siegel S.J. (2011). Poly Lactic-co-Glycolic Acid (PLGA) as Biodegradable Controlled Drug Delivery Carrier. Polymers.

[52] Koerner J., Horvath D., Herrmann V.L., MacKerracher A., Gander B., Yagita H., Rohayem J., Groettrup M. (2021). PLGA-particle vaccine carrying TLR3/RIG-I ligand Riboxxim synergizes with immune checkpoint blockade for effective anti-cancer immunotherapy. Nat. Commun..

[53] Cao J., Choi J.-S., Oshi M.A., Lee J., Hasan N., Kim J., Yoo J.-W. (2019). Development of PLGA micro- and nanorods with high capacity of surface ligand conjugation for enhanced targeted delivery. Asian J. Pharm. Sci..

[54] Jones K.S. (2008). Biomaterials as vaccine adjuvants. Biotechnol. Prog..

[55] Sharp F.A., Ruane D., Claass B., Creagh E., Harris J., Malyala P., Singh M., O’Hagan D.T., Pétrilli V., Tschopp J. (2009). Uptake of particulate vaccine adjuvants by dendritic cells activates the NALP3 inflammasome. Proc. Natl. Acad. Sci. USA.

[56] Joshi V.B., Geary S.M., Salem A.K. (2013). Biodegradable Particles as Vaccine Delivery Systems: Size Matters. AAPS J..

[57] Lin G., Wang J., Yang Y.-G., Zhang Y., Sun T. (2023). Advances in dendritic cell targeting nano-delivery systems for induction of immune tolerance. Front. Bioeng. Biotechnol..

[58] Baranov M.V., Kumar M., Sacanna S., Thutupalli S., Van Den Bogaart G. (2021). Modulation of Immune Responses by Particle Size and Shape. Front. Immunol..

[59] Askarizadeh M., Esfandiari N., Honarvar B., Sajadian S.A., Azdarpour A. (2023). Kinetic Modeling to Explain the Release of Medicine from Drug Delivery Systems. ChemBioEng Rev..

[60] Thejass P., Kuttan G. (2007). Immunomodulatory activity of Sulforaphane, a naturally occurring isothiocyanate from broccoli (*Brassica oleracea*). Phytomedicine.

[61] Orsi R., Funari S., Soares A., Calvi S., Oliveira S., Sforcin J., Bankova V. (2000). Immunomodulatory action of propolis on macrophage activation. J. Venom. Anim. Toxins.

[62] Thwe P.M., Amiel E. (2018). The role of nitric oxide in metabolic regulation of Dendritic cell immune function. Cancer Lett..

[63] Adediran E., Arte T., Pasupuleti D., Vijayanand S., Singh R., Patel P., Gulani M., Ferguson A., Uddin M., Zughaier S.M. (2025). Delivery of PLGA-Loaded Influenza Vaccine Microparticles Using Dissolving Microneedles Induces a Robust Immune Response. Pharmaceutics.

[64] Bagwe P., Bajaj L., Gala R.P., D’Souza M.J., Zughaier S.M. (2022). Assessment of In Vitro Immunostimulatory Activity of an Adjuvanted Whole-Cell Inactivated Neisseria gonorrhoeae Microparticle Vaccine Formulation. Vaccines.

[65] Gala R.P., Popescu C., Knipp G.T., McCain R.R., Ubale R.V., Addo R., Bhowmik T., Kulczar C.D., D’sOuza M.J. (2016). Physicochemical and Preclinical Evaluation of a Novel Buccal Measles Vaccine. AAPS PharmSciTech.

[66] Novitskiy S.V., Ryzhov S., Zaynagetdinov R., Goldstein A.E., Huang Y., Tikhomirov O.Y., Blackburn M.R., Biaggioni I., Carbone D.P., Feoktistov I. (2008). Adenosine receptors in regulation of dendritic cell differentiation and function. Blood.

[67] Furuta K., Onishi H., Ikada Y., Masaki K., Tanaka S., Kaito C. (2023). ATP and its metabolite adenosine cooperatively upregulate the antigen-presenting molecules on dendritic cells leading to IFN-γ production by T cells. J. Biol. Chem..

[68] Pacheco R., Martinez-Navio J.M., Lejeune M., Climent N., Oliva H., Gatell J.M., Gallart T., Mallol J., Lluis C., Franco R. (2005). CD26, adenosine deaminase, and adenosine receptors mediate costimulatory signals in the immunological synapse. Proc. Natl. Acad. Sci. USA.

[69] Casanova V., Naval-Macabuhay I., Massanella M., Rodríguez-García M., Blanco J., Gatell J.M., García F., Gallart T., Lluis C., Mallol J. (2012). Adenosine Deaminase Enhances the Immunogenicity of Human Dendritic Cells from Healthy and HIV-Infected Individuals. PLoS ONE.

