# Supplementary material for: Investigating the In Vitro Immunomodulatory Potential of Microparticulate β-L-Adenosine in Particulate Vaccine Candidates
Source: Vaccines (Basel). 2026 Feb 27;14(3):215. doi: 10.3390/vaccines14030215 (PMC13030022; doi:10.3390/vaccines14030215)
Supplement: Supplementary file 1 [file vaccines-14-00215-s001.zip › vaccines-4129860-supplementary.pdf]

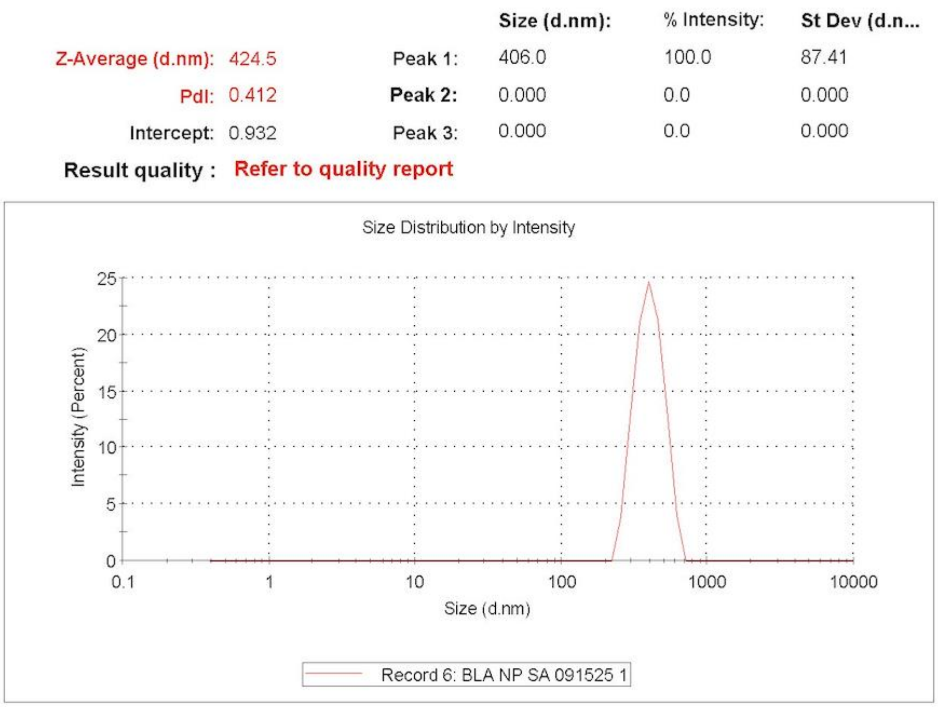

Supplementary Figure 1: Graphical representation of particle size of BLA MP.

Supplementary Table 1: Dose in micrograms per well in each treatment groups.

COMBINATIONS	TREATMENT GROUP	DOSE IN MICROGRAM PER WELL
Controls	Antigen MPs Alone (Example: H1N1, Zika, Measles, Canine Covid, Gonorrhea)	100
Controls	BLA MP	100
Controls	Adjuvant MPs Alone (Example: Alum, MF59)	100
Binary	Antigens MPs + Adjuvant MPs (Example: Zika MP + Alum MP; H1N1 MP + MF59 MP)	100 + 50
Binary	Antigen MPs + BLA MPs (Example: Measles MP + BLA MP)	100 + 50
Ternary	Antigen MPs + Adjuvant MPs + BLA MPs (Example: Gonorrhea MP + Alum MP + BLA MP)	100 + 25 +25

2.A. 2.B. 2.C.

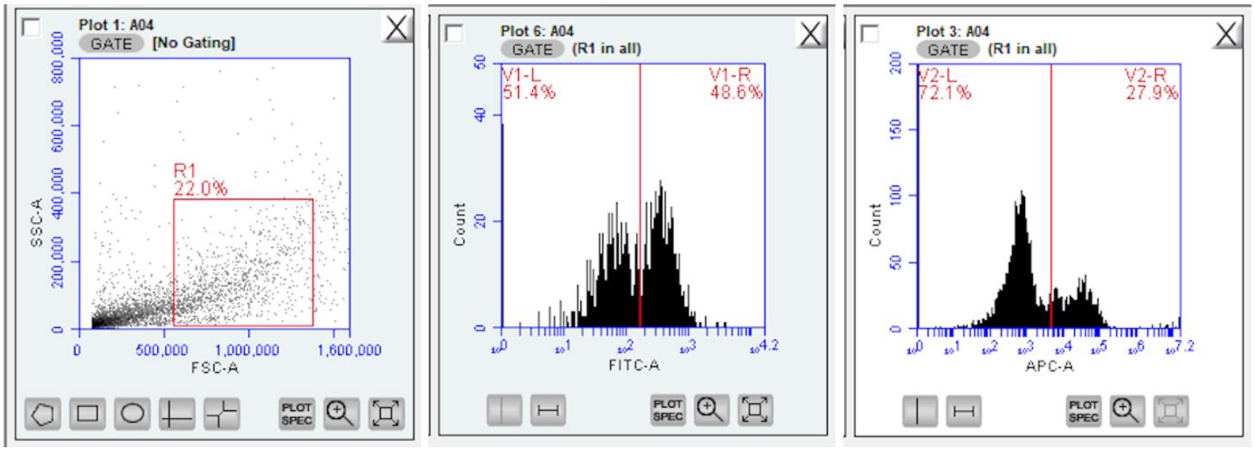

Supplementary Figure 2: Flow cytometry gating strategy for all experiments in this study. Figure 2. A. Scatter plot showing side scattering (SSC-A) and forward scattering (FSC-A) of the cells that are selected based on their size and granularity. The selection (R1) includes 22% of events from a given sample to analyze the fluorescence intensity. Figure 2.B. shows the histogram of FITC-A fluorescence intensity of a given sample. The events are divided into left and right panel where left indicates lower FITC-A expression and right indicates higher FITC-A expression in the selected population. Figure 2.C. shows the histogram of APC-A fluorescence intensity of a given sample. The events are divided into left and right panel where left indicates lower APC-A expression and right indicates higher APC-A expression in the selected population.
